# Massive lower gastrointestinal bleeding due to pseudoaneurysm of the femoral artery following buttock gunshot injury: A case report

**DOI:** 10.1016/j.ijscr.2024.110803

**Published:** 2024-12-29

**Authors:** Saman Sheikhi, Babak Mansourian, Aysa Karimi, Alireza Shakerpour, Ali Faegh, Fatemeh Nafarzadeh

**Affiliations:** a Department of Surgery, Shahid-Madani Hospital, School of Medicine, Alborz University of Medical Sciences, Karaj, Iran; b Radiology Department, Imam-Khomeini Hospital Complex, School of Medicine, Tehran University of Medical Sciences, Tehran, Iran; c School of Medicine, Alborz University of Medical Sciences, Karaj, Iran; d Student Research Committee, School of Medicine, Alborz University of Medical Sciences, Karaj, Iran

**Keywords:** False aneurysm, Gastrointestinal hemorrhage, Deep femoral artery, Gunshot wounds, Trauma

## Abstract

**Introduction:**

Arterio-enteric fistula is one of the extremely rare complications of penetrating trauma.

**Case presentation:**

A 27-year-old male presented to the emergency department with a gunshot to the right buttock. Initial assessments revealed a left femoral shaft fracture, the right buttock as the bullet inlet and the medial portion of the left thigh as an outlet, with no other significant findings. He underwent external fixation for the left femoral fracture. On the seventh day of admission, the patient experienced two episodes of massive melena and hypovolemic shock. The patient underwent an upper endoscopy without any remarkable findings. Also, we performed an emergent laparotomy to find the source of upper gastrointestinal bleeding (GIB); however, no evidence of upper GIB was found. Then, the patient underwent CT angiography, which subsequently identified a deep femoral artery pseudoaneurysm. Also, we performed a colonoscopy to investigate the source of GIB, revealing an orifice on the rectal wall. The patient underwent open surgery for a pseudoaneurysm. During surgery, a tract from the pseudoaneurysm of the deep femoral artery to the rectum was discovered, leading to the ligation of the deep femoral artery branch. After recovery from the operation and completion of the orthopedic treatment, the patient was discharged in an appropriate condition.

**Clinical discussion:**

Traumatic arterio-enteric fistulas can present with fatal gastrointestinal bleeding, requiring crucial investigations and proper imaging evaluations.

**Conclusion:**

In case of new-onset massive melena during hospitalization, upper GIB should always be considered. However, in penetrating trauma patients, repeating CT angiography should be considered.

## Highlights

• A deep femoral artery pseudoaneurysm due to a gunshot resulted in an arterio-enteric fistula.
• It presented seven days after admission with massive gastrointestinal bleeding.
• An exploratory laparotomy failed to identify the source of bleeding.
• CT angiography confirmed the presence of a deep femoral artery pseudoaneurysm.

## 1 Introduction

The buttock constitutes the lateral half of the lower-most sagittal zone of the torso, characterized by a high density of vital structures above and below the peritoneum in the pelvis [1]. Evidence indicates a significant frequency of life-threatening visceral and vascular injuries in patients with penetrating trauma to the buttock [2]. The pelvic anatomy predisposes individuals to significant complications or death following such injuries, regardless of the presence of vascular, abdominal, or pelvic signs [3]. One notable complication of penetrating trauma to the buttock is vascular injury, particularly involving the femoral arteries. [4]. Although penetrating trauma to the buttock is uncommon, it can occasionally lead to the development of a pseudoaneurysm. Post-injury, a hematoma may form in the tissue surrounding the artery, eventually developing a fibrous wall [5]. In such cases, arterio-enteric fistula can be developed, which can lead to fatal GIB. However, this is one of the most infrequent complications of penetrating trauma, with less than 30 reported cases during the past 25 years [6].

This report discusses a 27-year-old male who presented with a gunshot injury and subsequently developed massive gastrointestinal bleeding due to a deep femoral pseudoaneurysm and a fistula tract to the rectum seven days after sustaining a gunshot wound to the right buttock. The patient was successfully treated with ligation of the left deep femoral artery. The present study has been reported in line with the Surgical Case Report (SCARE) guideline [7].

## 2 Case presentation

**Fig. 1:**
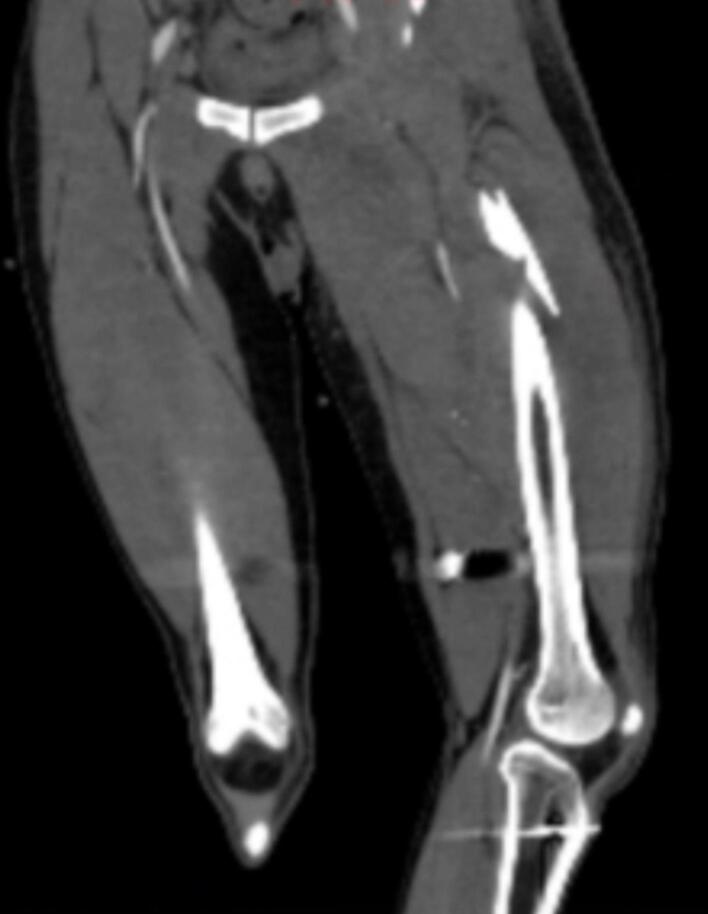
First-day computed tomography. Initial imaging indicated a left femoral bone shaft fracture without any evidence of pseudoaneurysm or fistula.

A 27-year-old male presented to our trauma center with a gunshot injury to the right buttock. Upon arrival, his vital signs were stable, his consciousness level was normal, and there were no signs of active external bleeding. Despite the history of multiple bullets fired at the scene of the accident, the patient's physical examination revealed the trajectory of only one bullet entered from the right buttock and exited from the distal and medial part of the left thigh. Also, a remarkable tenderness was found in the proximal part of the left thigh. Other physical examinations, including a digital rectal exam, were normal. Also, focused assessment with sonography in trauma (FAST) revealed no evidence of free fluid. According to the stable vital signs, he underwent initial computed tomography (CT) scans due to the high-energy trauma, revealing a significant left femoral shaft fracture and surrounding hematoma in the left adductor muscles (Fig. 1). According to the suspected history of multiple gunshots, we also performed computed tomography angiography, which was normal without any pathologic findings.

Initial laboratory tests revealed a hemoglobin level of 10.5 without any drop in repeated complete blood counts. Other laboratory tests were normal. The patient underwent external fixation of the left femoral bone. On the seventh day of hospitalization, he experienced two episodes of massive melena and hypotension (Systolic blood pressure = 80 mmHg), prompting an emergency surgical consultation. The patient reported two episodes of bloody stools and a huge amount of blood clots defecation, indicating massive upper GIB. After resuscitation and clinical suspicion of a stress ulcer, an emergent upper endoscopy was performed without any evidence of upper GIB.

According to the suspected history of multiple gunshot-related injuries and hypovolemic shock, he was taken to the operating room for an exploratory laparotomy. Before starting the surgery, a jugular central venous access was obtained for sufficient resuscitation, and the massive transfusion protocol was started. Due to a decrease in blood pressure despite the resuscitation, gastrostomy was performed; however, no evidence of gastric ulcer or bleeding was found. We found a massive amount of blood clots surrounding the rectum, colon, and ilium; however, no evidence of active bleeding was found. Also, the pelvic floor was clear on examination; however, the peritoneal reflection was not opened according to the critical condition to reduce the time of operation. The surgical team hypothesized possible injury of the rectal wall, so sigmoid colostomy placement was performed.

**Fig. 2:**
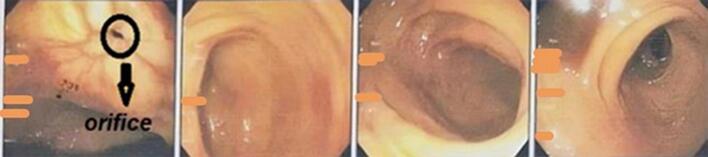
Rectal wall orifice, which was detected by colonoscopy.

**Fig. 3:**
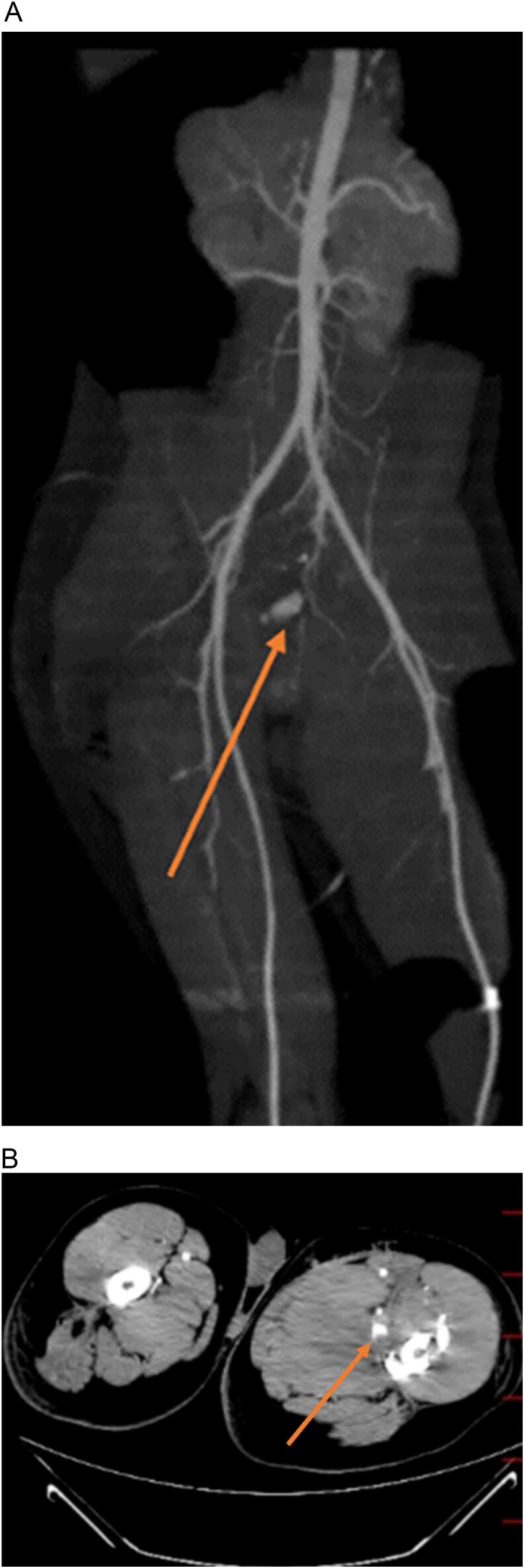
CT angiography revealed a pseudoaneurysm of the deep femoral artery. A: Coronal view; B: Axial view.

Post-operatively, with stabilized vital signs with sufficient resuscitation, we repeated CT angiography and a pseudoaneurysm of the deep femoral artery was identified (Fig. 3). Also, an emergent colonoscopy was performed to find the possible source of lower GIB, which revealed no mucosal bleeding in the gastrointestinal tract. However, an orifice was found in the rectal wall, with less than 5 cm distance from the anal verge, along with evidence of mild ischemic colitis (Fig. 2). After surgery, the patient experienced massive hematochezia, but no evidence of bleeding from the stoma was found. However, the vital signs were stable.

Due to the lack of interventional radiologist in our center, the patient underwent open surgery 48 h later. During the procedure, a clear tract from the pseudoaneurysm site to the rectum was observed, and the branch of the deep femoral artery was ligated. Post-operatively, the patient's symptoms were improved, and his vital signs stabilized. Ultimately, after completing orthopedic treatment for the fracture, he was discharged in good general condition with advice for follow-up regarding colostomy closure in an elective setting.

## 3 Discussion

The present study introduced a patient with a gunshot injury with a right buttock inlet and distal part of the left thigh outlet, causing an arterio-enteric fistula of the deep femoral artery to the rectal wall despite normal initial CT angiography, which was detected by repeated CT angiography.

Penetrating trauma to the gluteal region accounts for approximately 2–3 % of all penetrating injuries [8]. However, the mortality rate can be as high as 4–11 %, comparable to that of penetrating abdominal trauma [9,10]. Such trauma is primarily caused by gunshot and stab wounds and may result in various injuries, including rectal injuries, pelvic fractures, and, less frequently, vascular injuries [11]. The specific location of the trauma on the buttock also influences the likelihood of significant vascular injury [2]. Traumatic arterio-enteric fistulas are predominantly observed following penetrating trauma, with less than 30 reported cases documented in the past 25 years. These fistulas may present acutely or in a delayed manner with upper or lower gastrointestinal bleeding. A thorough clinical examination, supplemented by necessary imaging, is crucial for detecting such injuries [6]. Aorto-gastric, aorto-esophageal, and superior mesenteric-duodenal fistulas were reported in the limited studies; however, rectal fistulas are extremely rare. James et al. reported one case with a fistula of the inferior rectal artery to the rectum and another case of the gluteal artery-rectal fistula which both were managed endovascularly [6]. According to the limited number of reported cases, the diagnosis of the aorta-enteric fistula in such cases is still challenging. However, the standard process is usually performing CT-angiography before operation [6]. In our cases, due to the fact that the first upper endoscopy did not reveal the bleeding source, and the patient's condition was critical due to unstable vital signs and the presence of stage four hypovolemic shock, we decided to transfer the patient to the operating room before performing CT angiography.

Most of the recently reported cases were managed by endovascular procedures. Gel foam and coil embolization by selected angiography were used to manage arterio-rectal aneurysms [6]. However, due to the lack of interventional radiology facilities at our center, we performed aneurysm ligation by an open procedure.

## 4 Conclusion

We presented a case of a patient with a deep femoral artery pseudoaneurysm resulting from a prior gunshot wound, which developed a fistula tract to the rectum, leading to massive gastrointestinal bleeding. The patient was successfully treated with ligation of the artery. However, according to the normal initial CT angiography without any evidence of perforation of the colon's surrounding vessels, it is still unclear what is the supposed cause of the arterio-enteric fistula. This report underscores the necessity for proper management, follow-up protocols, and physician education regarding penetrating gluteal trauma with vascular injuries that may result in pseudoaneurysms and further complications. Patients exhibiting concerning symptoms, such as gastrointestinal bleeding, may benefit from early imaging follow-up with ultrasound or CT angiography to rule out pseudoaneurysm.

## Author contribution

**Saman Sheikhi**: Conceptualization, Methodology, and Supervision **Babak Mansourian**: Investigation, Resources, and Data Curation **Aysa Karimi**: Writing-Original Draft **Alireza Shakerpour**: Writing-Original Draft **Ali Faegh and Fatemeh Nafarzadeh**: Writing - Review & Editing.

## Ethical approval

This case study was exempt from ethical approval as informed consent was obtained directly from the patient for using images and publications. The Ethics Committee waived ethical approval at our institution.

## Guarantor

Babak Mansourian.

## Research registration number

N/A.

## Disclosure statement

While preparing this work, the authors used Microsoft copilot to improve readability and language. After using this tool/service, the authors reviewed and edited the content as needed and took full responsibility for the publication's content.

## Funding

The authors declare that they did not receive any funding for this study.

## Conflict of interest statement

All authors confirm that there is no conflict of interest.
